# Preparation and Evaluation of Directly Compressible Orally Disintegrating Tablets of Cannabidiol Formulated Using Liquisolid Technique

**DOI:** 10.3390/pharmaceutics14112407

**Published:** 2022-11-08

**Authors:** Ekapol Limpongsa, Peera Tabboon, Thaned Pongjanyakul, Napaphak Jaipakdee

**Affiliations:** 1 College of Pharmacy, Rangsit University, Pathum Thani 12000, Thailand; 2 Division of Pharmaceutical Technology, Faculty of Pharmaceutical Sciences, Khon Kaen University, Khon Kaen 40002, Thailand; 3 Center for Research and Development of Herbal Health Products, Khon Kaen University, Khon Kaen 40002, Thailand

**Keywords:** cannabinoids, disintegrating tablets, orodispersible tablets, liquid vehicles, dissolution enhancement

## Abstract

This study demonstrated the implementation of a liquisolid technique to formulate directly compressible orally disintegrating tablets (ODTs). Cannabidiol (CBD), a hydrophobic cannabinoid, was prepared as a liquisolid powder using microcrystalline cellulose–colloidal silicon dioxide as a carrier–coating material. Different liquid vehicles differing in their volatility, hydrophilicity, and viscosity were investigated. Each of the CBD–ODTs comprised CBD liquisolid powder (10 mg CBD), superdisintegrant, flavors, lubricant, and filler. The physical mixture (PM) ODT was prepared as a control. Ethanol-based ODTs (CBD–EtOH–ODTs) had comparable tablet properties and stability to CBD–PM–ODTs. ODTs with nonvolatile-vehicle-based liquisolid powder had lower friability but longer disintegration times as compared with CBD–PM–ODTs and CBD–EtOH–ODTs. Compression pressure influenced the thickness, hardness, friability, and disintegration of the ODTs. With a suitable compression pressure to yield 31-N-hardness-ODTs and superdisintegrant (4–8%), CBD–ODTs passed the friability test and promptly disintegrated (≤25 s). Times to dissolve 50% of CBD–PM–ODTs, CBD–EtOH–ODTs, and nonvolatile-vehicle-based CBD–ODTs were 10.1 ± 0.7, 3.8 ± 0.2, and 4.2 ± 0.4–5.0 ± 0.1 min, respectively. CBD–EtOH–ODTs exhibited the highest dissolution efficiency of 93.5 ± 2.6%. Long-term and accelerated storage indicated excellent stability in terms of tablet properties and dissolution. Nonvolatile-vehicle-based CBD–ODTs exhibited a higher percentage of remaining CBD. This study provides useful basic information for the development of ODT formulations using a liquisolid technique application.

## 1. Introduction

Solid dosage forms for oral administration are the most widely used and acceptable formulations due to their high stability, ease of manufacturing, small-size packaging, cost-effectiveness, convenience, and noninvasiveness [1]. Recent technologies in oral solid formulations have focused on the ease of administration and medication adherence enhancement, viz., those that disintegrate promptly within the mouth without the need for chewing or drinking water. Such systems include orally disintegrating tablets (ODTs). ODTs, also known as orodispersible tablets or fast disintegrating tablets, are patient-friendly novel formulations with the ability to deliver a large number of active pharmaceutical ingredients (API) to different groups of patients, in particular those who have swallowing dysfunction or dysphagia, as well as pediatric, geriatric, and psychiatric patients [2,3,4,5,6]. Previous reports have demonstrated that the orodispersible formulations of antipsychotic API yielded significantly larger adherence rates and patient preference in psychiatric patients. The ease of drug administration improvement reduces the treatment burden on the patients and also the caregivers [7]. The inclusion of orodispersible formulations in the European Pharmacopoeia and the commercial availability of these formulations emphasizes the significance of the orodispersible formulations as promising drug delivery systems.

An orally disintegrating tablet (ODT) is an uncoated tablet containing API or medicinal substances. It disintegrates instantly, usually within a matter of seconds, when placed upon the tongue, and becomes a solution or a suspension upon contact with the existing saliva in the oral cavity before being swallowed. According to the United States Food and Drug Administration (US FDA), ODTs require a disintegration time of within 30 s [8]. The API released from the ODTs can be aimed to treat locally in the oral cavity, or systematically by permeating through the oral mucosa or the gastrointestinal mucosa after swallowing [4]. ODTs have demonstrated the ability to conquer the problems related to oral absorption. They are also claimed to be an attractive alternative for API, with a first-pass metabolism [9]. 

Numerous methods have been utilized to fabricate the ODTs—compression, sublimation, phase transition, extrusion, molding, etc. Amongst these methods, direct compression is a principally preferential manufacturing process due to its superiorities, i.e., (1) simple and cost-efficient process, (2) availability of manufacturing and packaging equipment, (3) availability of excipients, and (4) ability to transfer into large-scale or industrial-scale manufacturing [6].

The rapid tablet disintegration of ODTs is able to yield a rapid onset of action. Nevertheless, for Biopharmaceutics Classification System (BCS) class II API, the dissolution, and hence the absorption, may still be limited due to their limited water solubility properties. It is well-known that API solubility regulates the dissolution performance of API from a solid formulation. The strategy for the dissolution enhancement of BCS class II API formulated into ODTs has grown in importance since approximately 40% of the APIs in development are poorly water-soluble compounds [10,11]. One of the promising dissolution enhancement approaches utilized in orodispersible formulations is the liquisolid technique. A “liquisolid technique” is designated for the transformation of liquid medicament into free-flowing and nonadherent solid powder. Solid API is generally turned into a liquid solution by dissolving with nonvolatile liquid vehicles or a mixture of nonvolatile and volatile vehicles. This resulting API solution, the so-called liquid medicament, is then absorbed/adsorbed onto the porous solid particles—carriers and coating materials—before being further manufactured into several solid dosage forms [12,13,14]. The efficacy of the liquisolid technique on the dissolution enhancement, as well as the bioavailability improvement, of several BCS class II APIs has been demonstrated [13,14,15,16,17,18]. Khan and coworkers [11] demonstrated that liquisolid compacts yielded a better dissolution performance improvement of BCS class II API than those prepared with a solid dispersion technique. It was recently reported that the utilization of the liquisolid technique successfully enhanced the dissolution behaviors of cannabinoids—delta-9-tetrahydrocannobinol, CBD, and cannabinol—from the compacts containing *Cannabis sativa* L. crude extract. Liquisolid formulations yielded an improvement in the dissolution efficiency at 120 min of 10–52, 11–41, and 61–405 times those of CBD, cannabinol, and delta-9-tetrahydrocannobinol, respectively [13]. A combination of liquisolid systems with ODTs for the delivery of BCS class II API has been reported. Koteswari et al. [19] investigated fast-disintegrating tablets of lamotrigine using the liquisolid technique. Polyoxyethylene sorbitan monolaurate (P20), microcrystalline cellulose (MCC), and colloidal silicon dioxide (CSD) were used as a carrier, coating material, and nonvolatile vehicle, respectively. The effects of different superdisintegrants—sodium starch glycolate (SSG) and crospovidone—were investigated. Moqbel et al. [20] demonstrated that the ODTs of chlorzoxazone could be successfully formulated with either co-processed superdisintegrants or the liquisolid technique. Both methods were competent for fabricating ODTs with acceptable properties and drug release improvement. Egla et al. [21] developed the zolmitriptan ODTs using the liquisolid technique. MMCC, CSD, and propylene glycol were used as a carrier, coating material, and liquid vehicle, respectively. The effects of different superdisintegrants were evaluated. The results demonstrate that crospovidone (5% *w*/*w*) was the best superdisintegrant yielding the fastest disintegration, as well as the superior drug release behavior, when compared to directly compressible ODTs. 

Cannabidiol (CBD) is a major non-psychoactive cannabinoid found in Cannabis species. Its medical utilization has grown due to the broad range of therapeutic potentials—antipsychotic, analgesic, antiseizures, antianxiety, anti-inflammatory, antioxidant, etc. [22,23,24]. Similar to other cannabinoids, CBD is a lipophilic (calculated log octanol/water partition coefficient of 8) BCS class II API. Its low water solubility (0.005 mg/mL, 37 °C) has been demonstrated [13,14]. The development of CBD in the form of orodispersible tablets has been executed. Ethicann Pharmaceuticals has developed cannabinoid-based CBD together with delta-9-tetrahydrocannabinol orodispersible tablets using Zydis technology, which is a platform based on the freeze-drying of tablet matrix components. This cannabinoid-based Zydis tablet has been used in preclinical studies on muscle spasticity treatment [23]. Vlad et al. [25] recently reported the development of CBD ODTs using a two-level full factorial design. The effects of formulation compositions, namely, poloxamer 407 concentration, type of co-processed excipient (Prosolv ODT G2 and Prosolv EasyTab sp), and type of superdisintegrant (croscarmellose (CCS) and soy polysaccharides), were demonstrated. According to this study, the optimized formulation showed a cumulative CBD released of nearly 100% at 30 min.

At present, no previous work has been described regarding CBD orodispersible formulations formulated using liquisolid systems. Additionally, limited investigations have demonstrated the effects of the types of liquid vehicles on the characteristics of the liquisolid powder-based ODTs. Accordingly, the present study aimed to investigate the CBD ODTs formulated using the liquisolid technique fabricated with direct compression. The effects of different nonvolatile liquid vehicles—diethylene glycol monoethyl ether (DEGEE), propylene glycol (PG), polyethylene glycol 400 (PEG), oleoyl macrogolglycerides (OM), caprylocaproyl macrogolglycerides (CM), and P20—used to prepared CBD liquisolid powder on the ODTs’ properties were evaluated. CBD was first prepared as a liquisolid system before being further formulated into ODTs. The CBD ODTs based on the physical mixture, as a control, and absolute ethyl alcohol (EtOH) formulations were also prepared. The effects of superdisintegrants—SSG and CCS—on the ODT properties were investigated. The physical and chemical stability of the fabricated CBD ODTs were also determined. 

In this study, MCC and CSD at the excipient ratio (R ratio) of 10:1 were used as carrier and coating materials. The term “carriers” refer to a solid particle possessing high specific surface area and thus liquid absorption capability due to the porous structure, enabling the liquid medication’s absorption/adsorption into its structure. Coating materials are extremely fine powder materials with excessive specific surface areas and adsorptive abilities. The coating materials will coat/cover the wet carrier, adsorb excess liquid, and turn the system into a free flowing powder [12,26]. The characteristics and capability of MCC and CSD in liquisolid systems have been reported [12,13,15,17].

## 2. Materials and Methods

### 2.1. Materials

The cannabidiol (CBD, CBD isolate, 99%) was received as a gift from the Medical Cannabis Research Institute, College of Pharmacy, Rangsit University (Mueang, Pathum Thani, Thailand). Microcrystalline cellulose (MCC; Avicel PH102) and croscarmellose sodium (CCS, Ac-Di-Sol) were received from Onimax Co., Ltd. (Bangkok, Thailand). Colloidal silicon dioxide (CSD, Aerosil 200) was received from Maxway Co., Ltd. (Bangkok, Thailand). Absolute ethyl alcohol (EtOH) was obtained from QRëC (Auckland, New Zealand). Caprylocaproyl macrogolglycerides (CM, Labrasol, Gattefossé SAS (Saint-Priest, French)), diethylene glycol monoethyl ether (DEGEE, Transcutol P, Gattefossé SAS (Saint-Priest, French)), oleoyl macrogolglycerides (OM, Labrafil M 1944 CS, Gattefossé SAS (Saint-Priest, French)), mannitol USP/EP (Mannogem EZ spray-dried mannitol), sodium starch glycolate (SSG, Explotab, JRS Pharma), and sodium stearyl fumarate (Lubripharm, SPI Pharma) were sourced from Rama Production, Co., Ltd. (Bangkok, Thailand). Glycerin and propylene glycol (PG) were supplied by RCI Labscan Ltd. (Bangkok, Thailand). Polyethylene glycol 400 (PEG) and polyoxyethylene sorbitan monolaurate (Polysorbate 20, P20) were purchased from Sigma-Aldrich (Missouri, MO, USA) and AppliChem GmbH (Darmstadt, Germany), respectively. Formic acid was obtained from KemAus (Cherrybrook, Australia). HPLC-grade methanol and acetonitrile were purchased from Fisher Scientific (Loughborough, UK). Spray-dried lactose (FlowLac 100) was provided by Meggle GmbH & Co. KG (Wasserburg am Inn, Germany). Stevioside or stevia powder was received from Krungthepchemi Co., Ltd. (Bangkok, Thailand). Menthol and peppermint oil were supplied by Thai-China Flavours and Fragrances Industry Co., Ltd. (Bangkok, Thailand). All chemicals were used as received.

### 2.2. Preparation of CBD Liquisolid Powder and CBD–EtOH Powder

CBD liquisolid powders based on different liquid vehicles—DEGEE, PG, PEG, OM, CM, and P20—were prepared using the formulation and method described recently by Tabboon et al. [14]. Briefly, 2 g of CBD isolate was dissolved with 8 g of liquid vehicles. The resulting solution was further dispersed with 69.1 g of MCC, followed by 6.9 g of CSD. For CBD powder prepared with EtOH (CBD–EtOH powder), the CBD–EtOH solution (2:8 ratio by weight) was dispersed with MCC (69.1 g), and then CSD (6.9 g). The residual EtOH was removed using a hot air oven (45 °C, 30 min). The obtained CBD liquisolid powder and CBD–EtOH powder were stored in the desiccator for further processing.

### 2.3. Preparation of CBD ODTs

The compositions of all investigated CBD ODT formulations are demonstrated in Table 1. All CBD ODT formulations were designed to comprise 10 mg CBD per tablet with a total tablet weight of 503 mg. To prepare CBD ODTs from CBD–EtOH powder and CBD liquisolid powder, the CBD powder was homogeneously mixed with flavored mannitol, stevioside, superdisintegrant, and spray-dried lactose (if necessary) (composition reported in Table 1) for a period of 5 min each using a cube mixer (ERWEKA GmbH, Langen, Germany). In the last stage of powder mixing, sodium stearyl fumarate was added and mixed for 5 min. In the case of the conventional physical mixture formulation, CBD–PM–ODTs, the CBD isolate powder was homogenously mixed with MCC using a geometric dilution technique for 10 min before mixing with other ingredients. The powder mixture (100.6 g, 200-tablet batch size for each formulation) was compressed into tablets using a Shimadzu hydrostatic press (Shimadzu Corporation, Kyoto, Japan) housed with a 13 mm round, flat-face tablet mold. Different compression pressures—namely, 2, 3.4, and 4.9 MPa (equivalent to 20, 35, and 50 kgf/cm^2^, respectively), and a compression pressure yielding tablet hardness of 31 ± 2 N—were investigated. The effects of superdisintegrant types (CCS and SSG) and concentrations (0, 4, and 8% *w*/*w*) were also determined. The obtained CBD ODTs were kept in a desiccator for further evaluation.

### 2.4. Powder Flowability

The flowability of the CBD ODT powder mixture was assessed using the methods for the powder flow test described in the United States Pharmacopeia (USP)—namely, flow rate, angle of repose, and Carr’s compressibility index [27]—as well as the angle of slide. The angle of slide is the flowability test method commonly used for liquisolid systems [28]. Each experiment was performed in triplicate.

The rate of powder flow through an orifice was examined using a flowability tester with funnel attachment and electronic balance (Copley model BEP2, Copley Scientific Ltd., Nottingham, United Kingdom). The tested powder mixture (~10 g) was consistently poured through a funnel (open ∅ 8 mm). The time taken by the given powder mass to flow was recorded and the powder flow rate in terms of the mass per time flowing from a funnel was computed.

(1) $$\mathrm{Flow}\;\mathrm{rate}\;=\frac{\mathrm{Powder}\;\mathrm{weight}\;\left(g\right)}{\mathrm{Flow}\;\mathrm{time}\;\left(s\right)}$$

The angle of repose was evaluated using the fixed height cone method. Briefly, a glass funnel was secured with its tip positioned at a given height (*H*) above a graph paper placed onto a smooth horizontal surface. The tested powder mixture (~5 g) was constantly poured through the funnel until the apex of the conical pile reached the tip of the funnel, generating a fixed-height conical pile. The mean radius (*r*) of the conical pile base was recorded and the angle of repose (*θ*) was computed using Equation (2).

(2) tan θ=H/r

Carr’s compressibility index was determined using the tapped and bulk densities of powders. Briefly, the bulk density was determined by pouring 25.0 g of the tested CBD powder mixture into a 100 mL graduated cylinder. The initial volume occupied by the powder was noted and used to compute a bulk density (g/mL). The cylinder containing the powder was tapped manually, 1.5 inches in height, 200 times. The tapped volume of the powder was recorded and used to compute the tapped density (g/mL). Carr’s compressibility index was calculated using Equation (3). The flowability of the CBD ODT powder mixture was evaluated according to USP criteria [27].

(3) $$\mathrm{Carr}\text{’}s\;\mathrm{compressibility}\;\mathrm{index}=\frac{\mathrm{Tapped}\;\mathrm{density}\;-\;\mathrm{Bulk}\;\mathrm{density}\;}{\mathrm{Tapped}\;\mathrm{density}\;}\times 100$$

To determine the angle of slide, approximately 4 g of the CBD ODT powder mixture was deposited on one end of a surface-polished stainless-steel plate (10 × 15 cm^2^). This end was delicately lifted till the CBD powder mixture began to fall or slide. The angle between the horizontal surface and the stainless-steel plate that caused the slide of the powder was noted. 

### 2.5. Evaluation of CBD-ODTs

#### 2.5.1. Tablet Properties

The tablet properties—namely, tablet weight, thickness, hardness, friability, and disintegration—of the prepared CBD ODTs were evaluated according to the USP [27]. 

The weight and thickness of ten CBD ODTs were determined individually using an analytical balance (ME204E, Mettler-Toledo GmbH, Greifensee, Switzerland) and thickness gauge (Teclock model SM-112, Teclock Co., Ltd., Nagano, Japan), respectively. The average and standard deviation values were calculated.

The hardness was evaluated using a tablet hardness tester (VanKel Model 40-2100 VK200, VanKel, North Carolina, USA), as was the friability using a tablet friability tester (VanKel Model 45-2200, VanKel, North Carolina, USA). Thirteen CBD ODTs (>6.5 g) were weighed (*W*_1_) and loaded into the drum of an apparatus, which was subsequently set to rotate at 25 rpm for 100 revolutions (4 min). Afterward, the CBD ODTs were removed, dedusted, and reweighed (*W*_2_). The percentage of weight loss denoted as the percentage of friability was calculated using Equation (4).

(4) $$\mathrm{Percentage}\;\mathrm{of}\;\mathrm{friability}=\left(\frac{\;W_{1}-W_{2}\;}{W_{1}}\right)\times 100$$

The disintegration time was evaluated using a basket rack 6-tube assembly disintegration tester (Model ZT-324, Erweka America Inc., Edison, NJ, USA). Deionized water (800 mL) warmed at 37 ± 0.5 °C was used as a tested medium. Each CBD ODT was positioned into the basket with a disc. The time taken by the CBD ODTs to disintegrate completely and be liberated from the screen of the basket was recorded as the disintegration time.

#### 2.5.2. CBD Content Uniformity

Each tablet of CBD ODTs was weighed and dispersed with 250 mL of methanol. The mixture was magnetically stirred for 12 h to extract and completely dissolve the CBD before being filtered through a 0.45 μm syringe filter. The CBD content was analyzed using an HPLC assay.

#### 2.5.3. Wettability

The wettability of the CBD ODTs was investigated in terms of wetting time and water absorption ratio using the method described recently [13]. Shortly, each CBD ODT was weighed (*W_d_*) and placed on the filter paper moistened with a color solution (2% *w/v* amaranth, 5 mL). The wetting time, referring to the time needed to wet the tablet completely, was recorded. The weight of the wet tablet (*W_w_*) was subsequently determined. The water absorption ratio was computed as follows.

(5) $$\mathrm{Water}\;\mathrm{absorption}\;\mathrm{ratio}\;\left(\%\right)=\frac{\left(W_{w}-W_{d}\right)}{W_{d}}\times 100$$

#### 2.5.4. In Vitro Dissolution 

The dissolution of CBD from the CBD ODTs was investigated using the paddle apparatus (Hanson Research model 72RL, Hanson Research Corp., Chatsworth, CA, USA). The test conditions were 500 mL of simulated saliva fluid (SSF), pH 6.8, comprising 1% *w/v* P20 maintained with 50 ± 2 rpm and a temperature of 37 ± 0.5 °C. At a predetermined time, aliquots of 10 mL were removed and filtered through a 0.45 μm syringe filter. The filtrate was quantified for the CBD via the HPLC method. The cumulative amount of dissolved CBD was computed and plotted against time. The time to dissolve 50% (T_50%_) and the dissolution efficiency, expressed as the rectangular area percentage determined from the area under the dissolution curve up to 120 min, of CBD from the CBD ODTs formulations were quantified [16,29]. To compare the dissolution profiles with the control (CBD–PM–ODTs), the model-independent similarity factor (*f*_2_) was computed as per Equation (7) [15,16].

(6) $$\mathrm{Dissolution}\;\mathrm{efficiency}=\frac{\int_{0}^{t}y\times dt}{y_{100}\times t}\;$$

(7) $$f_{2}=50\times log[\frac{1}{\sqrt{1+\left(\frac{1}{n}\right)\sum_{t=1}^{n}{\left(R_{t}-T_{t}\right)}^{2}}}\times 100]\;$$

where *n* is the number of sampling points, *R_t_* is the percent of reference dissolved (CBD–PM–ODTs), and *T_t_* is the percent of test dissolved.

#### 2.5.5. Physical and Chemical Stability 

The stability of CBD ODTs under long-term and accelerated conditions was investigated. The recently prepared CBD ODTs were separately positioned in 24-well polystyrene plates (with internal diameter of 15 mm). The CBD ODTs-containing plate was sealed in a laminated aluminum sachet and kept at 5 ± 3 °C (long-term) and 30 ± 2 °C with relative humidity (RH) of 75 ± 5% (accelerated). After 4 months of storage, the aged CBD ODTs were withdrawn and evaluated for the percentage of CBD remaining in each tablet, as well as tablet properties, wettability, and dissolution. The change in CBD dissolution behaviors of aged ODTs, in comparison with fresh ODTs, was also assessed in terms of the similarity factor (*f*_2_), which was calculated using the Equation (7) [15,16].

### 2.6. HPLC Assay

The quantity of CBD was determined using the chromatographic method [14]. Briefly, the HPLC system of an Agilent series 1260 coupled with an autosampler and diode array detector was used. The analytical column was an Agilent ZORBAX Eclipse Plus C_18_ column (100 × 4.6 mm, 3.5 μm) operated at 45 °C. The mobile phase was acetonitrile:water (70:30 volume ratio) containing 0.1% formic acid operated with a 1.5 mL/min flow rate. The total chromatographic runtime was 10 min, and the CBD retention time was 6.0 min. The chromatographic method exhibited acceptable linearity over the investigated concentration, which ranged from 2 to 80 μg/mL, with a coefficient of determination of >0.9995.

### 2.7. Statistical Analysis

One-way analysis of variance (ANOVA) with Tukey’s post hoc test operated via the SPSS program for Windows software (Version 17.0, Released 2008, SPSS Inc., Chicago, IL, USA) was utilized for the statistical analysis. The statistical significance was set at *p* < 0.05.

## 3. Results and Discussion

To prepare the ODTs formulated with the liquisolid systems, different CBD liquisolid powders were fabricated using MCC–CSD as the carrier–coating materials. Additionally, the CBD–EtOH powder was also prepared to study the effect of the volatile vehicle EtOH. The CBD isolate was firstly dissolved in the liquid vehicles to yield a CBD concentration of 20% *w*/*w*. The loading factor value of 0.145 was used irrespective of the liquid vehicle types to rule out the influence of the loading factor value on the requisite quantity of the carrier–coating materials. All of the resulting CBD powders were white, dry-looking, and non-adherent. The good flowability of these CBD liquisolid powders has been reported [14]. 

The CBD ODTs composed of 10 mg CBD in the form of liquisolid powder, CBD–EtOH powder, or CBD isolate to yield CBD loading per tablet were manufactured using direct compression. Other necessities were superdisintegrant, flavoring agents, lubricant, and filler (if necessary). To improve mouthfeel and palatability, stevioside and peppermint oil–menthol-flavored spray-dried mannitol were used as sweeteners and flavoring agents, respectively. Sodium stearyl fumarate was used as a water-soluble lubricant. The tablet size of all the CBD ODT formulations was kept constant by using spray-dried lactose as a filler to adjust the final tablet weight to 503 mg. For comparative purposes, the conventional physical mixture formulation of CBD ODTs (CBD–PM–ODTs) was also prepared as a control. To ensure that all CBD ODTs were fabricated under comparable compression conditions, the powder mixture for each CBD ODTs formulation was die-filled and hand compressed with the designed compression pressure. The success of ODT preparation via the direct compression approach generally depends on the use of suitable formulation compositions and compression pressures to yield a fast-disintegrating tablet matrix with sufficient hardness and acceptable friability. Therefore, the effects of superdisintegrant addition and compression pressure on the disintegration time and hardness of CBD ODTs were firstly investigated.

### 3.1. Influence of Superdisintegrants

The effect of superdisintegrants—CCS (0 and 4%) and SSG (0, 4, and 8%)—on the disintegration of CBD ODTs was determined. Different CBD ODTs formulated with four different CBD powders containing different liquid vehicles, namely, EtOH, DEGEE, PG, and P20, were preliminarily utilized. The selected liquid vehicles differ in their physicochemical properties, i.e., volatility, hydrophilicity, and viscosity. For the CBD–EtOH formulation, EtOH was used as a volatile solvent vehicle, to allow the deposition of CBD onto the carrier–coating surface after the removal of EtOH by oven-drying. In the case of the CBD liquisolid powder, the nonvolatile vehicle–CBD solution was deposited together onto the carrier–coating surface. Thus, the physical properties of the nonvolatile vehicle were predicted to influence not only the dissolution behavior but also the tablet properties. 

All of the CBD ODTs were directly compressed with 4.9 MPa compression pressure. The resulting ODTs were white circular tablets with a smooth and shiny flat surface and a tablet weight in the range of 504 ± 2–506 ± 1 mg. The friability values of all investigated formulations were lower than 1% (0.02–0.18%), signifying the adequate mechanical durability of the CBD ODTs. For uncoated tablets, such as ODTs, high friability results in an unacceptable loss of API content during downstream processing (e.g., packaging), handling, and storage. The excessive loss of API content may affect the therapeutic efficacy due to sub-potency. Additionally, a defective tablet appearance may generate uncertainty regarding tablet quality in patients [30]. Their thickness ranged from 3.49 ± 0.02 to 3.58 ± 0.01 mm for CBD ODTs without nonvolatile liquids—CBD–PM–ODTs and CBD–EtOH–ODTs—and from 3.22 ± 0.01 to 3.29 ± 0.02 mm for CBD ODTs contained nonvolatile liquids—CBD–DEGEE–ODTs, CBD–PG–ODTs, and CBD–P20–ODTs. 

**Figure 1:**
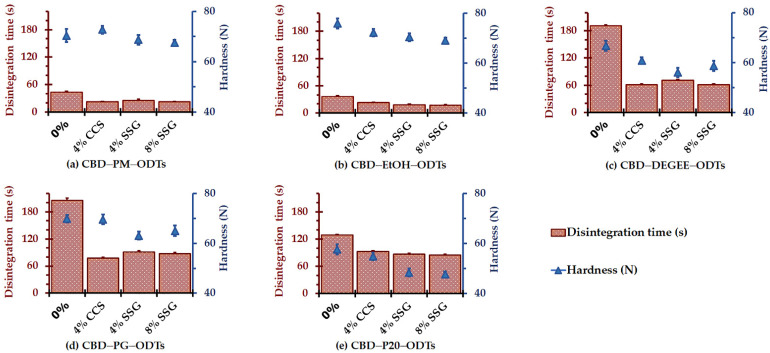
Influence of superdisintegrants on tablet hardness and disintegration time of 4.9-MPa-compressed CBD ODTs (mean ± SD, *n* = 6).

Figure 1 summarizes the hardness and disintegration time of the CBD ODTs as a function of superdisintegrants. It can be seen that CBD–PM–ODTs and CBD–EtOH–ODTs without superdisintegrant addition disintegrated within 44 ± 2 s and 37 ± 2 s, respectively. The addition of CCS (4%) and SSG (4% and 8%) significantly shortened the disintegration time to the range of 22 ± 2–26 ± 2 s for CBD–PM–ODTs, and 17 ± 1–23 ± 1 s for CBD–EtOH–ODTs. The hardness of CBD–PM–ODTs and CBD–EtOH–ODTs was in the range of 67.6 ± 1.1–72.7 ± 1.5 N and 69.1 ± 1.6–75.9 ± 2.1 N, respectively.

The rapid disintegration of CBD–PM–ODTs and CBD–EtOH–ODTs, regardless of superdisintegrant content, was associated with the MCC properties. MCC is a commonly known direct compression binder due to its excellent dry-binding properties [31,32]. MCC also has a self-disintegrating property due to its high hydrophilicity and hygroscopicity, as well as its high intraparticle porosity properties, which permit rapid water uptake into both the intraparticle and interparticle spaces [32,33]. These phenomena induce pore pressure and thus pore enlargement. Together with the interparticulate attraction perturbation caused by the adhesive force between water and adjacent particles generated during water uptake, the CBD ODTs were eventually broken down into their constituent particles [33].

For the CBD ODTs prepared from nonvolatile liquid-based CBD liquisolid powder, the lengthening of the disintegration time compared to those of the control and CBD–EtOH–ODT formulations was observed. For those without superdisintegrants, they disintegrated within 129 ± 5–205 ± 5 s. This may be associated with the greater cohesiveness of the tablet matrix mass strengthened by the vehicles. DEGEE, PG, and P20 are nonvolatile liquids with functional groups that are available to form hydrogen bonds. DEGEE is an ether alcohol with low viscosity (5 mPa·s) and low polarity (dielectric constant of 14.1) [34]. PG is a diol compound with two hydroxyl groups in its structure. It is a lightly viscous (58 mPa·s), semipolar liquid with a dielectric constant of 32.1. P20 is a polymeric surface active agent comprising PEGylated sorbitan with 20 poly(ethylene glycol) units. P20 is a highly viscous liquid (400 mPa·s) with a hydrophilic–lipophilic balance (HLB) of 16.7 [31,35]. These nonvolatile liquids that physically adsorbed/absorbed onto the MCC’s surface possibly served as a binder by forming a liquid bridge between the MCC particles, facilitating and strengthening the adhesive force, e.g., hydrogen bonding, between them. Additionally, either the hydrophilicity, viscosity, or in combination, of the nonvolatile liquids might influence the hydration and thus the rate of water uptake into the intraparticle and interparticle space. It was recently reported that the hydration of nonvolatile liquid, e.g., P20, was related to the increase in the dynamic viscosity [36]. These phenomena might delay the rate of water uptake and thus the disintegration. 

The incorporation of 4–8% superdisintegrant, CCS or SSG, substantially decreased the time to disintegrate to the ranges of 61–71, 77–91, and 85–92 s for CBD–DEGEE–ODTs, CBD–PG–ODTs, and CBD–P20–ODTs, respectively. The abilities of both CCS and SSG as superdisintegrants are associated with their excellent water uptake, wicking, and swelling capacities [37,38,39]. The use of these superdisintegrants at higher concentrations than the recommended used concentrations (0.5–5.0% *w*/*w* and 2.0–8.0% *w*/*w* for CCS and SSG, respectively) could induce disintegration prolongation due to the formation of a viscid gel layer impeding water penetration and thus slowing down the disintegration. The effects of CCS and SSG, alone or in combination, on the disintegration of orodispersible tablets have been well investigated [37,40,41]. Koteswari et al. [19] reported that SSG exhibited better efficiency in terms of the disintegrating and dissolution rate improvement of lamotrigine fast-disintegrating tablets prepared with the liquisolid technique as compared with crospovidone. According to Desai et al. [37], an investigation of superdisintegrant combinations in the ODTs revealed that the total quantity of superdisintegrant needed for an individual or combination of two superdisintegrants to yield the acceptable outcome responses was comparable. Therefore, the use of a superdisintegrant, either alone or in combination with two compounds, was recommended. 

It is known that high concentrations of SSG extend the disintegration time due to the viscid gel layer formation, which presumably retards the water uptake into the CBD ODTs [37]. Nevertheless, the comparable efficacies of 4% SSG and 8% SSG when compared with 4% CCS in terms of the disintegration time of CBD ODTs were observed. This was partially caused by the effects of SSG on the hardness of CBD ODTs, especially those containing nonvolatile liquid vehicles. As can be seen for all CBD ODTs, their hardness decreased with SSG concentrations. A decrease in the compressibility with the superdisintegrant concentration in the formulation has been reported [40]. 

### 3.2. Influence of Compression Pressure 

The effect of compression pressure (2, 3.4, and 4.9 MPa) on the disintegration, as well as the thickness and hardness of CBD ODTs, was determined. The CBD ODTs could be fabricated successfully by using all of the investigated compression pressures, nevertheless, not all of the applied pressures yielded robust CBD ODTs. For CBD–PM–ODTs, CBD–EtOH–ODTs, and CBD–DEGEE–ODTs, the compression pressures of ≤2 MPa yielded highly friable tablets (>5% friability), thus failing the friability test. Interestingly, CBD–PG–ODTs and CBD–P20–ODTs passed the friability test irrespective of the applied compression pressure. 

**Figure 2:**
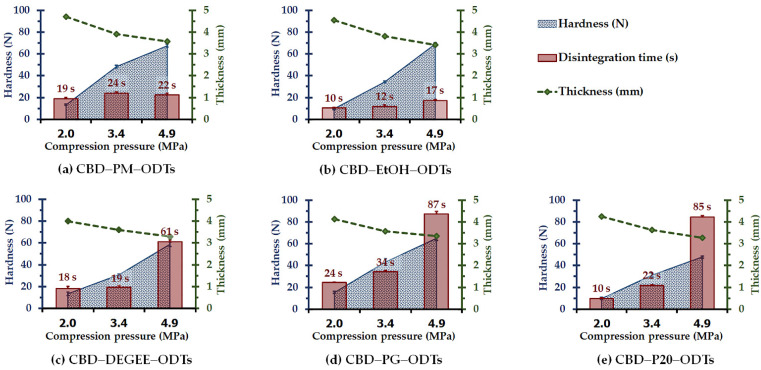
Influence of compression pressure on hardness, disintegration, and thickness of various CBD ODTs containing 8% SSG (mean ± SD, *n* = 6).

Figure 2 presents the disintegration time, as well as thickness and hardness, as a function of the compression pressure of five different CBD ODTs. As can be seen for all CBD ODTs, the hardness increased with the compression pressures. This was attributable to the decrease in the interparticulate volume and void space when a higher compression pressure was applied, as indicated by the tablet thickness values. Additionally, it is known that MCC undergoes plastic deformation under compression pressure, and the CBD ODTs are composed of 68.7% MCC. The higher the compression pressure, the higher the surface area composed of the joined MCC particles [17,42,43]. The smaller space between the particles, as well as the larger contact surface area, could promote and strengthen the intermolecular bonding among the particle surfaces, i.e., hydrogen bonding by the MCC, resulting in a more compact and cohesive structure of CBD ODTs. It should be noted that the changes in thickness and hardness of CBD ODTs were nearly proportionate to the applied compression pressure increase. It is also interesting to remark that the hardness of the CBD–P20–ODTs formulation seems to be less sensitive to the applied compression pressure when compared with the other formulations. This is in agreement with the previous report. Lam et al. [44] recently reported the effect of a nonvolatile liquid, polysorbate 80, on the hardness of naproxen Liqui-Tablets fabricated from Liqui-Pellets. They found that the hardness of the Liqui-Tablet reduced with the concentration of polysorbate 80 in the formulation.

In terms of disintegration time, for the CBD ODTs without nonvolatile vehicles, they disintegrated rapidly; within 19–22 s and 10–17 s for CBD–PM–ODTs and CBD–EtOH–ODTs, respectively, irrespective of the applied compression pressure. For CBD ODTs containing nonvolatile vehicle, the high compression pressure of 4.9 MPa dramatically lengthened the disintegration time. This is attributed to the more coherent and compact structure facilitated by the nonvolatile vehicles and high compression pressure. These led to a slower rate of water uptake into and through the CBD ODTs, delaying the intermolecular bonding replenishment with the entering of water molecules, the wicking and swelling of the superdisintegrant, and thus the tablet breakdown process. This indicated that for ODTs formulated with a nonvolatile liquid vehicle containing liquisolid powder, the applied compression pressure should be sufficient to yield a robust tablet with a prompt disintegration ability, i.e., within 30 s, upon contact with an aqueous medium. It should be noted that for CBD liquisolid powder-based ODTs, the tablet hardness of approximately 31 ± 2 N could provide satisfying outcomes in terms of friability and disintegration. As presented in Figure 2, by using the compression pressure of 3.4 MPa, CBD–DEGEE–ODTs with tablet hardness of 30.7 ± 1.0 N disintegrated within 19 ± 2 s, and CBD–P20–ODTs with tablet hardness of 31.1 ± 1.5 N disintegrated within 22 ± 1 s. 

### 3.3. CBD ODTs Properties: Influence of Liquid Vehicles

Eight formulations of CBD ODTs based on CBD powder, prepared with different vehicles, and the control formulations were fabricated. EtOH and different nonvolatile vehicles—nonvolatile liquid solvents with different dielectric constant values (DEGEE, PEG, and PG) and nonvolatile liquid surfactants with different HLB values (OM, CM, and T20)—were investigated. The SSG at 8% *w*/*w* was used as a superdisintegrant. Other formulation compositions (Table 1) were kept constant. 

#### 3.3.1. Flowability of CBD ODT Bulk Powder Mixture 

The powder mixture designed for direct compression must be flowable to permit consistent feeding through the hopper into the tablet die cavity, ensuring the uniformity of the weight and active content. The bulk powder mixture of CBD ODT formulations was therefore assessed for its density and flowability characteristics, and the results are summarized in Table 2. All of the CBD ODT powder mixtures have a bulk density ranging from 0.32 ± 0.02 to 0.44 ± 0.03 and a tapped density ranging from 0.39 ± 0.02 to 0.55 ± 0.02. Their flow rate was in the range of 1.47 ± 0.04–1.77 ± 0.1 g/s. In compliance with USP [27], all of the CBD ODT formulations exhibited fair-aid not needing flow, with the Carr’s compressibility index range of 19.19 ± 2.87–20.16 ± 1.11. Concerning the angle of repose criteria, the powder mixture was classified as having good flow because of its angle of repose value being lower than 35°. In terms of the angle of slide, all of the CBD ODTs powder mixtures showed acceptable flowability due to their angle of slide values of less than 33° [13]. These results suggest that the CBD ODT powder mixture has sufficient flowability for tableting by the direct compression process. 

The acceptable flow property of the CBD ODT powder mixture was attributed to the free-flowing properties of the formulation compositions—MCC (68.7%), SSG (8%), spray-dried mannitol (5%), and spray-dried lactose (8%, for CBD–PM–ODTs and CBD–EtOH–ODTs) [10]. The comparable flowability of the CBD ODT powder mixture irrespective of the liquid vehicle type was attributable to the sufficient amounts of carrier–coating materials used in the CBD liquisolid systems to permit evidently non-adherent, dry, and flowable powder. The good and acceptable flowability of the liquisolid system-based powder mixture prepared using the optimal loading factor has been widely reported [13,14,15].

#### 3.3.2. Tablet Properties 

To investigate the influence of liquid vehicles on tablet properties, the CBD ODT was designed to have a comparable hardness of 31 ± 2 N by applying sufficient compression pressure (3–4 MPa). The tablet properties of the resulting CBD ODTs were evaluated, and the results are listed in Table 3. All CBD ODTs had tablet thicknesses ranging from 3.5 to 3.8 mm. The greater thickness of CBD–PM–ODTs and CBD–EtOH–ODTs is related to the higher solid mass and thus the volume of the tablets (*p* < 0.05). The hardnesses of CBD ODTs were comparable (*p* > 0.05) and within the designed hardness range. All CBD ODTs had acceptable friability of lower than 1%, which meets the official criteria for tablet friability [27]. It should be noted that the CBD ODTs formulated with CBD liquisolid powder exhibited a lower friability value than those of CBD–EtOH–ODTs and CBD–PM–ODTs, even though they had comparable hardness. This may be related to the binding property of nonvolatile liquid vehicles, connecting the particles through the liquid bridges. Additionally, nonvolatile liquid vehicles may also improve tablet plasticity, and thus reduce brittleness. The lower and more acceptable friability of nonvolatile vehicle-containing liquisolid tablets as compared to that of the conventional physical mixture formulation has been reported [44].

The disintegration test revealed that all CBD ODTs were disintegrated within 30 s, ranging from 16 ± 2 to 25 ± 2 s, which are within the criteria recommended by the US FDA. According to FDA guidance, the ODTs should exhibit in vitro disintegration times of no more than 30 s [8]. The rapid mechanical break-up of CBD ODTs into their constituent particles upon contact with the medium was the combined result of disintegrating compositions (SSG and MCC) as well as the suitable hardness. The slight but significantly longer disintegration times of CBD–PG–ODTs and liquid surfactant-containing CBD ODTs than those of CBD–EtOH–ODTs and CBD–PM–ODTs (*p* < 0.05) are attributable to the more coherent and compact structure enhanced by these nonvolatile vehicles. The influence of nonvolatile vehicles on tablet integrity enhancement has been reported [13,44]. 

The wettability of CBD ODTs was assessed by placing the ODTs on a wet surface, permitting the migration of water from its base to the top. All CBD ODTs demonstrated comparable wettability, in terms of wetting time and water absorption ratio (Table 3). These indicate that they have equivalent wettability irrespective of the vehicle type (*p* > 0.05). This is attributable to either the high and equal amounts of hydrophilic, water-soluble components, or both, in the formulation. 

The CBD content assay indicated the uniformity of CBD content irrespective of the formulations. The percentage of CBD in all CBD ODTs ranged between 97.2 ± 3.0 and 98.0 ± 3.9% (Table 4). 

All of the fabricated CBD ODTs had acceptable tablet properties in terms of friability, disintegration time, and content uniformity regardless of the liquid vehicle types. Nevertheless, for oromucosal formulations, such as ODTs, an undesirable taste of the formulation component would affect the palatability and thus patient acceptance and adherence. PEG is a bitter liquid with a slightly burnt taste, while polysorbates have a characteristic odor with a warm and bitter taste [31]. Concerning the palatability of the liquid vehicles, CBD–PEG–ODTs and CBD–P20–ODT formulations were not selected for further investigations.

#### 3.3.3. In Vitro Dissolution

The in vitro dissolution of the selected CBD ODTs under sink conditions was carried out using a paddle apparatus with 1% *w/v* P20 pH 6.8 SSF as a dissolution medium. The solubilities of CBD in pH 6.8 SSF and 1% *w/v* P20 pH 6.8 SSF at 37 °C, determined using the method previously described by Tabboon et al. [14], were found to be approximately 0.003 mg/mL and 1.3 ± 0.4 mg/mL, respectively. Because of the poor solubility in aqueous medium of CBD, 1% *w/v* P20 was added as a solubilizer to improve the solubility of CBD in an aqueous medium and to provide the sink condition for the dissolution test. The use of a surfactant to increase the API solubility in the dissolution medium is widely reported. The aqueous solution containing 1% *w/v* P20 was proven to be sufficient to provide the sink condition for performing the dissolution test of cannabinoids [13].

**Figure 3:**
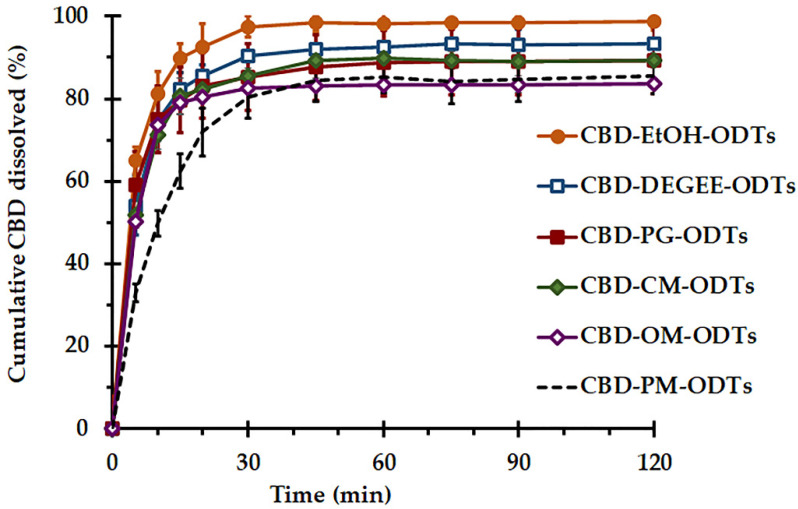
In vitro dissolution of CBD from CBD ODTs under sink conditions at 37 ± 0.5 °C (mean ± SD, *n* = 6). CBD ODTs were composed of 8% SSG and compressed at a sufficient compression pressure (3–4 MPa) to yield tablets with a hardness of 31 ± 2 N.

The influence of liquid vehicles on the dissolution of CBD ODTs is presented in Figure 3. All CBD ODTs presented similar dissolution patterns and reached the plateau within 30 min. CBD–PM–ODTs showed the slowest and most incomplete dissolution with 77.3 ± 4.6% dissolution efficiency (Table 4). The comparison of the dissolution profiles, performed using the similarity factor (*f*_2_), suggests that CBD–EtOH–ODTs and CBD liquisolid powder-based ODTs demonstrated dissimilar dissolution behaviors in comparison with CBD–PM–ODTs as their *f*_2_ values were lower than 50. CBD–OM–ODTs had the largest *f*_2_ of almost 50, whereas CBD–EtOH–ODTs yielded the smallest *f*_2_ value. In terms of time to dissolve 50% CBD (T_50%_), ODTs formulated with CBD liquisolid powder exhibited significantly shorter T_50%_ when compared to CBD–PM–ODTs, and CBD–PG–ODTs resulted in the fastest T_50%_ (*p* < 0.05) amongst the CBD liquisolid powder-based ODTs. A slight but significant improvement in the dissolution efficiency of the CBD liquisolid powder-formulated ODTs, except CBD–OM–ODTs, compared to CBD–PM–ODTs was observed (*p* < 0.05). Interestingly, compared with all CBD ODTs, CBD–EtOH–ODTs exhibited the fastest T_50%_ and highest dissolution efficiency (*p* < 0.05).

In accordance with the FDA, the percentage of API dissolved from ODT formulations should be at least 85% in 30 min [45]. Accordingly, CBD–PM–ODTs and CBD–OM–ODTs failed to comply with this requirement. The incomplete dissolution behavior of CBD–PM–ODTs may be related to the SSG behavior. The prompt water uptake and excessive swelling of SSG may hinder the CBD release. This swelling causes the buildup of a viscous gel layer acting as a barrier for CBD diffusion. In the case of CBD–OM–ODTs, its incomplete CBD dissolution was also associated with the low hydrophilicity (HLB~9) and viscid nature (75–95 mPa·s [31]) of OM, which might affect the amount of CBD available for dissolution. It has been demonstrated that the viscosity of liquid surfactants was raised with hydration, especially with a limited volume of an aqueous medium. This viscosity modification may induce pore-clogging and trap the CBD–OM solution inside the pores of MCC-CSD [36]. The incomplete dissolution of API from the ODTs prepared from surfactant-based liquisolid systems and containing high contents of superdisintegrants (5% SSG and 5% crospovidone) have been reported [19]. 

The greatest degree of CBD dissolution from CBD–EtOH–ODTs was simply achieved via surface area enlargement. By using EtOH as a solvent vehicle, CBD was able to deposit on both the inner and outer surfaces of MCC–CSD pores in a non-crystalline form after EtOH evaporation [14]. 

The slight, but significant, improvement in the dissolution behaviors of CBD ODTs formulated with liquisolid systems—CBD–DEGEE–ODTs, CBD–PG–ODTs, and CBD–CM–ODTs—compared to CBD–PM–ODTs was related to the surface area increment of CBD available for dissolution. This resulted from the adsorption/absorption of CBD in a non-crystalline state onto the MCC–CSD surface. It was recently demonstrated that, by using attenuated total reflection-Fourier transform infrared and X-ray diffraction, the CBD in the CBD liquisolid powder was structurally associated with liquid vehicles and lost crystallinity [14]. It was also claimed that a small amount of nonvolatile liquid vehicle presented on the MCC–CSD surface could enhance the saturated solubility (*C_s_*) of CBD in the microenvironment, either via the cosolvency mechanism (for liquid solvents) or via micellar solubilization (for liquid surfactants), as well as the wettability of the CBD–MCC–CSD surface [12,13,18,46,47]. For CBD–PG–ODTs, its improved dissolution compared to CBD–DEGEE–ODTs, CBD–CM–ODTs, and CBD–OM–ODTs was possibly associated with the solubilization potential of PG and its physical properties (semipolar solvent with moderate viscosity (58 mPa·s [31])). PG was reported to be an excellent solvent for CBD [14]. 

Tabboon et al. [14] recently demonstrated that, by using static diffusion cells, different liquid vehicles diversely influenced the in vitro diffusion of CBD from liquisolid systems. This was related to the physical properties (viscosity, hydrophilicity (dielectric constant), HLB) and surface-active properties of liquid vehicles. It is interesting to note that, here, the dissolution investigation with a large quantity of dissolution medium presented the least discriminative influence of liquid vehicles on the CBD ODTs’ dissolution performance. As stated in the Noyes–Whitney equation, the dissolution rate is in proportion to the surface area available for contact with the medium and the concentration gradient in the stagnant diffusion layer (Δ*C = C_s_* − *C*). Under sink conditions, in dissolution study, the CBD ODTs instantly disintegrated into their constituent particles once immersed in the medium. The CBD–PM–ODTs simply exposed the CBD isolate particles, while for CBD liquisolid powder-formulated ODTs, the large surface area of CBD located on the MCC–CSD surface was exposed to a large volume of medium. The abundant surface area available and the high concentration gradient resulting from the low CBD concentration in the bulk solution (*C*) accelerated the rate of CBD dissolution. This phenomenon might impede the effects of liquid vehicles on the microenvironment. This is in line with previous studies reporting that a more significant dissolution improvement of liquisolid tablets was noticed when smaller volumes of dissolution media were used [48,49].

It was recently reported by Vlad et al. [25] that the CBD ODTs were successfully developed to meet the FDA requirements using suitable superdisintegrant—soy polysaccharides (Emcosoy STS IP)—and components—poloxamer 407, co-processed excipient (Prosolv ODT G2), mannitol, sorbitol, and banana flavor. The fast disintegration due to suitable superdisintegrant and the use of poloxamer 407 might be the reason for the almost complete CBD dissolution within 30 min. However, it was noted that the higher value of poloxamer 407 demonstrated a negative impact on the crushing strength and CBD release. Additionally, the stability information of these previously established CBD ODTs has not been investigated yet.

### 3.4. Stability 

All the selected CBD ODTs were subjected to tests investigating the effect of aging on chemical stability and tablet properties. It has been demonstrated that cannabinoids were more susceptible to high storage temperatures [13,50]; therefore, CBD ODT formulations are considered to be drug products intended for storage in a refrigerator. The long-term and accelerated conditions for the stability test were 5 ± 3 °C and 30 ± 2 °C/75 ± 5% RH, respectively (Climatic Zone IVb [51]). 

**Figure 4:**
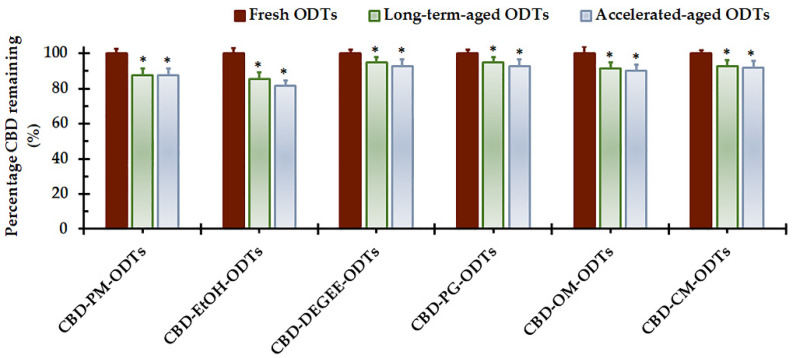
Percentage of remaining CBD in CBD ODTs stored under long-term (5 ± 3 °C) and accelerated (30 ± 2 °C/75 ± 5% RH) conditions for 4 months (mean ± SD, *n* = 3). The CBD ODTs were composed of 8% SSG and compressed under a sufficient compression pressure (3–4 MPa) to yield tablets with a hardness of 31 ± 2 N. * Significant decrease as compared to the fresh CBD ODTs (*p* < 0.05).

Figure 4 presents the percentages of CBD remaining in the CBD ODTs after four months of storage as compared to that of the fresh samples. It clearly shows that the percentage of remaining CBD in the four-month-old CBD ODTs was significantly lower than the initial values (*p* < 0.05) regardless of the storage conditions. After storage under long-term conditions for four months, the percentages of CBD remaining were 85.5–87.6% for CBD–PM–ODTs and CBD–EtOH-ODTs, and 91.6–95.0% for CBD liquisolid powder-based ODTs. Under accelerated conditions, the percentages of CBD remaining after four months of storage were 81.4–85.4% for CBD–PM–ODTs and CBD–EtOH–ODTs, and 90.3–92.9% for CBD liquisolid powder-based ODTs. CBD is very susceptible to oxidation and photodecomposition. CBD deterioration can also be induced by high temperatures, acid pH, and basic pH [24,52]. However, in this study, a comparable percentage of CBD loss in each CBD ODT formulation during storage under long-term versus accelerated conditions was observed (*p* > 0.05). This is in line with the previous report finding that the contents of remaining cannabinoids, including CBD, in cannabis extract–vegetable oil sublingual drops stored in the dark at 4 °C and 30 °C for three months were similar [53]. Recent investigations reveal that the solvent affects CBD stability. According to Fraguas-Sánchez et al. [52], an aqueous solution exhibited lower CBD stability than that of ethanolic solution. A slightly greater percentage of remaining CBD was likely to be observed with liquisolid powder-based CBD ODTs, whereas those of CBD–PM–ODTs and CBD–EtOH–ODTs were comparable (*p* > 0.05). The solubilization of CBD with a nonvolatile, viscous liquid before deposition onto porous MCC–CSD might decrease the available contact surface between CBD and harsh environments, e.g., oxygen and moisture. It has been claimed that the liquisolid system prepared with nonvolatile liquid is a promising alternative to enhance drug photostability in solid dosage forms due to the high refractive index and light diffraction ability of CSD [12,16]. 

The tablet properties of aged CBD ODTs were assessed, and the results are presented in Table 5. The four-month-old CBD ODTs, irrespective of storage conditions, exhibited comparable tablet properties to those of fresh ODTs (*p* > 0.05). Aged CBD ODTs showed acceptable friability values under 1%, although the trend of increase in friability values with aging was noticed. This might be associated with the water adsorption of the ODTs’ surface, owing to the highly hygroscopic and hydrophilic nature of the ingredients [54]. The adsorbed water molecules might disturb the interparticulate bonding across particle–particle interfaces, resulting in more friable ODTs. It should be noted that because of the low hardness and thus friable nature of the fabricated CBD ODTs, packaging considerations must be made. The primary packaging for the CBD ODTs should provide a sufficient mechanical barrier and moisture absorption/loss protection to preserve the tablet integrity, as well as stability, of the ODTs [6,45]. 

The in vitro dissolution analysis indicated the close similarity in the dissolution performance of aged and fresh CBD ODTs (Figure S1), as the values of similarity factor (*f*_2_) were higher than 50. These results indicate that the physical appearance and properties of CBD ODTs were not affected by aging. The stability of liquisolid tablets prepared with MCC–CSD upon storage has been widely reported [13,15,29,55].

## 4. Conclusions

This investigation demonstrated that the liquisolid approach can be successfully implemented to formulate the ODTs. The effects of different liquid vehicles on the tablet properties, dissolution behaviors, and stability of the ODTs were revealed for the first time. The utilization of a volatile vehicle, EtOH, enabled the deposition of CBD onto the MCC–CSD surface, similar to nonvolatile vehicle employment. CBD–EtOH powder-based ODTs exhibited comparable tablet properties and CBD stability, as compared to the physical mixture formulation. It should be noted that, for CBD ODTs, the limited improvement in the dissolution behaviors of CBD–EtOH powder-based and liquisolid powder-based ODTs, as compared to the physical mixture ODTs, was evidenced. This study provides information regarding the characteristics of EtOH powder-based and liquisolid powder-based ODTs.

## Figures and Tables

**Table 1:** General formulation compositions of CBD ODTs.

Ingredients	*% w/w*	CBD ODT Formulations (mg per Tablet)		
		Physical Mixture (CBD–PM–ODTs)	EtOH-Based System (Volatile Vehicle *)	Liquisolid Systems (Nonvolatile Vehicles **)
CBD	2.0	10	10	10
Liquid vehicle	8.0	0	40 *	40 **
MCC	68.7	345.5	345.5	345.5
CSD	6.9	34.5	34.5	34.5
Superdisintegrant ***	0, 4.0, 8.0	0, 20.1, 40.2	0, 20.1, 40.2	0, 20.1, 40.2
Flavored mannitol ****	5.0	25.2	25.2	25.2
Stevioside	1.0	5.0	5.0	5.0
Sodium stearyl fumarate	0.5	2.5	2.5	2.5
Spray-dried lactose to make		503	503	503

* EtOH was the volatile vehicle, which was dried out during the preparation process. ** Nonvolatile vehicles for liquisolid systems were PG, PEG, DEGEE, CM, OM, and T20, respectively. *** Superdisintegrants were CCS or SSG at the concentration of 0%, 4%, or 8% *w*/*w*. **** Flavored mannitol was a mixture of peppermint oil, menthol, and spray-dried mannitol (1:1:18 ratio by weight).

**Table 2:** Bulk density, tapped density, and flowability of CBD ODT bulk powder mixture.

Powder Mixture Formulations	Bulk Density (g/mL)	Tapped Density (g/mL)	Carr’s Compressibility Index	Flow Rate (g/s)	Angle of Repose (°)	Angle of Slide (°)
CBD–PM–ODTs	0.32 ± 0.02	0.39 ± 0.02	19.19 ± 2.87	1.69 ± 0.11	34.06 ± 1.17	28.00 ± 1.00
CBD–EtOH–ODTs	0.34 ± 0.01	0.42 ± 0.01	19.72 ± 1.05	1.77 ± 0.12	34.14 ± 1.05	26.67 ± 1.53
CBD–DEGEE–ODTs	0.44 ± 0.03	0.55 ± 0.02	19.27 ± 2.79	1.56 ± 0.12	34.28 ± 0.68	28.33 ± 0.58
CBD–PEG–ODTs	0.41 ± 0.01	0.51 ± 0.01	19.49 ± 1.36	1.61 ± 0.07	34.32 ± 0.54	28.67 ± 0.58
CBD–PG–ODTs	0.41 ± 0.01	0.51 ± 0.02	19.49 ± 1.36	1.69 ± 0.11	34.39 ± 0.94	28.00 ± 1.01
CBD–OM–ODTs	0.41 ± 0.01	0.51 ± 0.01	19.81 ± 1.30	1.54 ± 0.08	33.93 ± 1.09	29.00 ± 1.00
CBD–CM–ODTs	0.43 ± 0.02	0.53 ± 0.03	19.63 ± 1.23	1.47 ± 0.04	34.38 ± 0.11	27.67 ± 0.58
CBD–P20–ODTs	0.42 ± 0.01	0.53 ± 0.01	20.16 ± 1.11	1.56 ± 0.07	33.75 ± 0.69	28.67 ± 0.58

The SSG at 8% *w*/*w* was used as a superdisintegrant. Mean ± SD, *n* = 3.

**Table 3:** Influence of liquid vehicle on tablet properties and wettability of CBD ODTs.

ODT Formulations *	Thickness (mm) ^1^	Hardness (N) ^2^	Friability (%)	Disintegration Time (s) ^2^	Wetting Time (s) ^3^	Water Absorption Ratio (%) ^3^
CBD–PM–ODTs	3.7 ± 0.01 ^a^	30.9 ± 1.0 ^a^	0.68	16 ± 4 ^a^	36 ± 2 ^a^	193.2 ± 5.0 ^a^
CBD–EtOH–ODTs	3.8 ± 0.03 ^b^	31.2 ± 0.7 ^a^	0.68	16 ± 2 ^a^	33 ± 2 ^a^	186.8 ± 4.3 ^a^
CBD–DEGEE–ODTs	3.8 ± 0.03 ^b^	31.9 ± 1.0 ^a^	0.42	21 ± 3 ^a^	35 ± 2 ^a^	193.5 ± 8.2 ^a^
CBD–PEG–ODTs	3.6 ± 0.02 ^c^	32.1 ± 1.1 ^a^	0.25	20 ± 2 ^a^	32 ± 2 ^a^	198.6 ± 9.4 ^a^
CBD–PG–ODTs	3.6 ± 0.03 ^c^	31.2 ± 0.7 ^a^	0.30	24 ± 2 ^b^	35 ± 1 ^a^	192.0 ± 6.7 ^a^
CBD–OM–ODTs	3.5 ± 0.03 ^d^	31.9 ± 1.1 ^a^	0.29	25 ± 2 ^b^	33 ± 2 ^a^	184.9 ± 7.0 ^a^
CBD–CM–ODTs	3.5 ± 0.02 ^d^	31.9 ±1.0 ^a^	0.25	22 ± 3 ^b^	33 ± 2 ^a^	182.9 ± 6.6 ^a^
CBD–P20–ODTs	3.6 ± 0.03 ^c^	31.4 ± 1.6 ^a^	0.17	22 ± 3 ^b^	38 ± 1 ^b^	190.6 ± 8.2 ^a^

* The SSG at 8% *w*/*w* was used as a superdisintegrant. CBD ODTs were compressed with sufficient compression pressure (3–4 MPa) to yield the tablets with hardnesses of 31 ± 2 N. ^1^ *n* = 20, ^2^ *n* = 6, ^3^ *n* = 3, mean ± SD. ^a–c^ Means in the same column sharing a common superscript letter are not different (*p* > 0.05), as analyzed by one-way ANOVA and Tukey’s post hoc test.

**Table 4:** CBD content and in vitro dissolution parameters of CBD ODTs containing 8% SSG and having the hardness of 31 ± 2 N.

ODT Formulations	CBD Content (%)	In Vitro Dissolution		
		T_50%_ (min)	Dissolution Efficiency (%)	*f*_2_
CBD–PM–ODTs	97.7 ± 2.5 ^a^	10.1 ± 0.7 ^a^	77.3 ± 4.6 ^a^	N/A
CBD–EtOH–ODTs	97.2 ± 3.0 ^a^	3.8 ± 0.2 ^b^	93.5 ± 2.6 ^b^	35
CBD–DEGEE–ODTs	97.5 ± 2.1 ^a^	4.6 ± 0.6 ^c^	87.4 ± 2.1 ^c^	43
CBD–PG–ODTs	98.0 ± 3.9 ^a^	4.2 ± 0.4 ^b^	84.0 ± 1.1 ^c^	44
CBD–OM–ODTs	97.9 ± 4.0 ^a^	5.0 ± 0.1 ^c^	79.4 ± 1.0 ^a^	49
CBD–CM–ODTs	97.4 ± 2.0 ^a^	4.8 ± 0.3 ^c^	84.0 ± 3.2 ^c^	47

Mean ± SD, *n* = 6. ^a–c^ means in the same column without a common superscript letter are different (*p* < 0.05), as analyzed by one-way ANOVA and Tukey’s post hoc test. T_50%_ refers to the time required for dissolving 50% of CBD. Dissolution efficiency is the percentage of rectangular area under the dissolution curve up to 6 h. *f*_2_ refers to the similarity factor in comparison with CBD–PM–ODTs. N/A, not applicable.

**Table 5:** Tablet properties of 4-month-old CBD ODTs stored at 5 ± 3 °C (long-term) and 30 ± 2 °C/75 ± 5% RH (accelerated) conditions compared with fresh ODTs.

Formulations	Weight (mg) ^1^	Thickness (mm) ^1^	Hardness (N) ^2^	Friability (%)	Disintegration Time (s) ^3^	Wetting Time (s) ^3^	Water Absorption Ratio (%) ^3^	Dissolution Behaviors ^2^		
								T_50%_ (min)	Dissolution Efficiency (%)	*f*_2_
CBD–PM–ODTs										
Fresh ODTs	504 ± 2	3.7 ± 0.01	30.9 ± 1.0	0.65	16 ± 4	36 ± 2	193.2 ± 3.0	10.1 ± 0.7	77.3 ± 4.6	N/A
Long-term-aged ODTs	506 ± 2	3.7 ± 0.01	30.9 ± 1.0	0.77	19 ± 4	36 ± 2	184.7 ± 3.2	9.5 ± 0.2	79.8 ± 2.3	79
Accelerated-aged ODTs	505 ± 2	3.7 ± 0.02	31.2 ± 0.7	0.86	20 ± 4	36 ± 1	187.2 ± 2.5	10.5 ± 1.4	76.5 ± 3.2	93
CBD–EtOH–ODTs										
Fresh ODTs	505 ± 2	3.8 ± 0.03	31.2 ± 0.7	0.68	16 ± 2	33 ± 2	186.8 ± 4.3	3.8 ± 0.2	93.5 ± 2.6	N/A
Long-term-aged ODTs	503 ± 2	3.7 ± 0.02	31.7 ± 1.0	0.74	16 ± 2	33 ± 1	181.6 ± 3.1	4.6 ± 0.4	94.0 ± 2.4	71
Accelerated-aged ODTs	503 ± 3	3.7 ± 0.03	31.6 ± 0.7	0.75	15 ± 2	33 ± 2	182.8 ± 2.3	4.2 ± 0.3	92.2 ± 2.8	84
CBD–DEGEE–ODTs										
Fresh ODTs	504 ± 1	3.8 ± 0.03	31.9 ± 1.0	0.42	21 ± 3	35 ± 2	193.5 ± 3.2	4.6 ± 0.6	87.4 ± 2.1	N/A
Long-term-aged ODTs	505 ± 2	3.7 ± 0.02	31.6 ± 1.3	0.55	22 ± 2	33 ± 2	192.5 ± 2.7	4.6 ± 0.3	89.2 ± 2.9	83
Accelerated-aged ODTs	505 ± 1	3.7 ± 0.03	32.1 ± 0.8	0.59	24 ± 3	34 ± 2	186.9 ± 2.4	4.6 ± 0.1	86.5 ± 1.3	92
CBD–PG–ODTs										
Fresh ODTs	505 ± 1	3.6 ± 0.03	31.2 ± 0.7	0.30	24 ± 2	35 ± 1	192.0 ± 2.7	4.2 ± 0.4	84.0 ± 1.1	N/A
Long-term-aged ODTs	504 ± 2	3.6 ± 0.03	32.5 ± 1.1	0.45	23 ± 2	38 ± 2	193.5 ± 3.0	4.2 ± 0.4	85.2 ± 1.7	88
Accelerated-aged ODTs	504 ± 2	3.6 ± 0.03	31.9 ± 1.0	0.56	23 ± 3	37 ± 2	196.0 ± 2.2	4.3 ± 0.1	82.8 ± 1.9	91
CBD–OM–ODTs										
Fresh ODTs	505 ± 2	3.5 ± 0.03	31.9 ± 1.1	0.29	25 ± 2	33 ± 2	184.9 ± 3.0	5.0 ± 0.1	79.4 ± 1.0	N/A
Long-term-aged ODTs	506 ± 2	3.5 ± 0.01	31.2 ± 0.9	0.41	25 ± 2	31 ± 2	184.1 ± 2.5	4.9 ± 0.2	81.4 ± 2.7	83
Accelerated-aged ODTs	505 ± 1	3.5 ± 0.02	32.9 ± 1.2	0.48	28 ± 3	33 ± 2	180.0 ± 2.6	5.2 ± 0.4	78.6 ± 3.9	94
CBD–CM–ODTs										
Fresh ODTs	504 ± 2	3.5 ± 0.02	31.9 ± 1.0	0.25	22 ± 3	33 ± 2	182.9 ± 6.6	4.8 ± 0.3	84.0 ± 3.2	N/A
Long-term-aged ODTs	504 ± 1	3.5 ± 0.02	32.1 ± 1.0	0.38	21 ± 3	33 ± 1	185.2 ± 2.8	4.7 ± 0.2	86.7 ± 2.3	79
Accelerated-aged ODTs	505 ± 2	3.5 ± 0.02	32.2 ± 0.7	0.41	23 ± 3	33 ± 2	179.2 ± 2.9	5.0 ± 0.1	83.0 ± 3.5	91

All CBD ODTs were composed of 8% SSG and compressed at a sufficient compression pressure (3–4 MPa) to yield tablets with a hardness of 31 ± 2 N. ^1^ *n* = 20, ^2^ *n* = 6, ^3^ *n* = 3, mean ± SD. *f*_2_ refers to the similarity factor of 4-month-old CBD ODTs in comparison with their fresh ODTs. N/A, not applicable.

## Data Availability

The data are available on request.

## Supplementary Materials

The following supporting information can be downloaded at: https://www.mdpi.com/article/10.3390/pharmaceutics14112407/s1, Figure S1: Dissolution behaviors of aged as compared to fresh CBD ODTs after 4 months of storage at 5 ± 3 °C (long-term) and 30 ± 2 °C/75 ± 5% RH (accelerated) conditions (mean ± SD, *n* = 6): (a) CBD–PM–ODTs, (b) CBD–EtOH–ODTs, (c) CBD–DEGEE–ODTs, (d) CBD–PG–ODTs, (e) CBD–OM–ODTs, and (f) CBD–CM–ODTs.
