# Supplementary figures and images for: Preparation and Evaluation of Directly Compressible Orally Disintegrating Tablets of Cannabidiol Formulated Using Liquisolid Technique
Source: Pharmaceutics. 2022 Nov 8;14(11):2407. doi: 10.3390/pharmaceutics14112407 (PMC9695279; doi:10.3390/pharmaceutics14112407)

Figure S1.

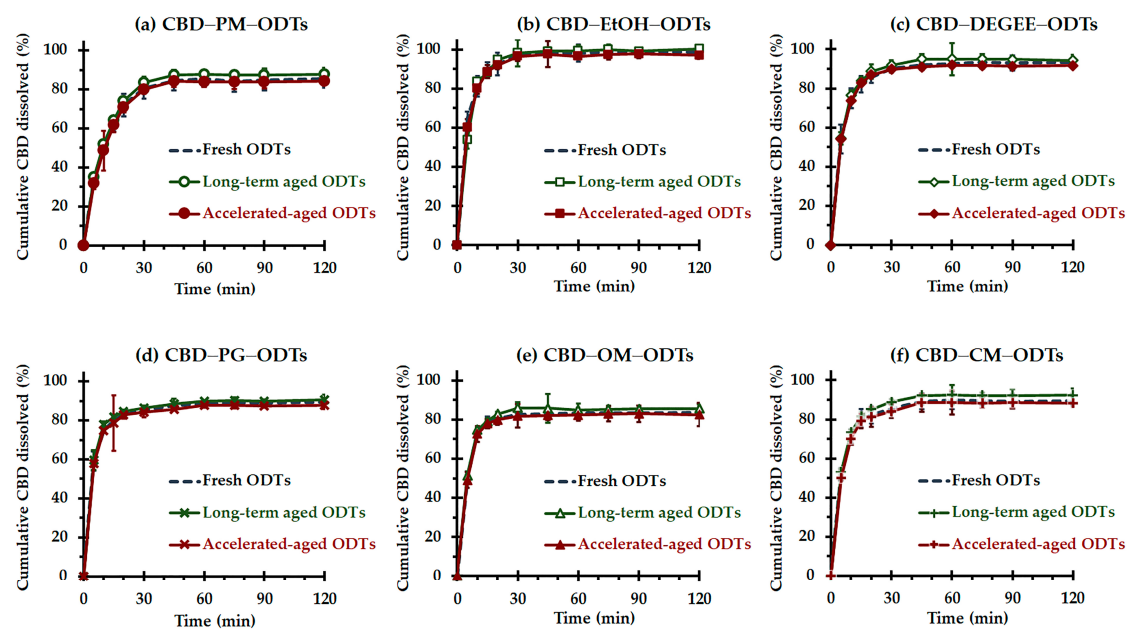

Supplement: Supplementary file 1 [file pharmaceutics-14-02407-s001.zip › pharmaceutics-1961145-supplementary.pdf]
